# Characteristics of Glucose Metabolism in Nordic and South Asian Subjects with Type 2 Diabetes

**DOI:** 10.1371/journal.pone.0083983

**Published:** 2013-12-31

**Authors:** Cecilie Wium, Hanne Løvdal Gulseth, Erik Fink Eriksen, Kåre Inge Birkeland

**Affiliations:** 1 Department of Endocrinology, Morbid Obesity and Preventive Medicine, Oslo University Hospital, Oslo, Norway; 2 Hormone Laboratory, Oslo University Hospital, Oslo, Norway; 3 Faculty of Medicine, University of Oslo, Oslo, Norway; University of Barcelona, Faculty of Biology, Spain

## Abstract

**Background:**

Insulin resistance and type 2 diabetes are more prevalent in people of South Asian ethnicity than in people of Western European origin. To investigate the source of these differences, we compared insulin sensitivity, insulin secretion, glucose and lipid metabolism in South Asian and Nordic subjects with type 2 diabetes.

**Methods:**

Forty-three Nordic and 19 South Asian subjects with type 2 diabetes were examined with intra-venous glucose tolerance test, euglycemic clamp including measurement of endogenous glucose production, indirect calorimetry measuring glucose and lipid oxidation, and dual x-ray absorptiometry measuring body composition.

**Results:**

Despite younger mean ± SD age (49.7±9.4 vs 58.3±8.3 years, *p = 0.001*), subjects of South Asian ethnicity had the same diabetes duration (9.3±5.5 vs 9.6±7.0 years, *p = 0.86*), significantly higher median [inter-quartile range] HbA_1c_ (8.5 [1.6] vs 7.3 [1.6] %, *p = 0.024*) and lower BMI (28.7±4.0 vs 33.2±4.7 kg/m^2^, *p<0.001)*. The South Asian group exhibited significantly higher basal endogenous glucose production (19.1 [9.1] vs 14.4 [6.8] µmol/kgFFM⋅min, p* = 0.003).* There were no significant differences between the groups in total glucose disposal (39.1±20.4 vs 39.2±17.6 µmol/kgFFM⋅min, *p = 0.99*) or first phase insulin secretion (AUC_0–8 min_: 220 [302] vs 124 [275] pM, p* = 0.35*). In South Asian subjects there was a tendency towards positive correlations between endogenous glucose production and resting and clamp energy expenditure.

**Conclusions:**

Subjects of South Asian ethnicity with type 2 diabetes, despite being younger and leaner, had higher basal endogenous glucose production, indicating higher hepatic insulin resistance, and a trend towards higher use of carbohydrates as fasting energy substrate compared to Nordic subjects. These findings may contribute to the understanding of the observed differences in prevalence of type 2 diabetes between the ethnic groups.

## Introduction

The prevalence of type 2 diabetes (T2D) varies between different ethnic groups, and is known to be high in South Asians (SA) in their countries of origin, particularly in urban areas, but also after migration to Western countries [1]. Several theories as to why SA are especially prone to insulin resistance and T2D have been proposed. Among these, the adipose tissue overflow hypothesis proposes that SA have smaller superficial subcutaneous adipose tissue compartments for fat storage compared to Western Europeans. Hence, in situations of energy excess, fat is deposited in the more metabolically active deep subcutaneous and visceral adipose tissue compartments [2]. The metabolic inflexibility theory proposes that the normal switch in energy substrate between high lipid oxidation in the fasting state and high glucose oxidation in the post-prandial state is impaired in T2D. This results in accumulation of intramuscular lipids and increased plasma glucose values [3]. A third suggested mechanism involves the theory that SA have lower resting energy expenditure (REE) than Western Europeans, and therefore are more prone to obesity and insulin resistance. This theory is disputed, where some argue that the lower REE is due to differences in body composition and not ethnicity [4], [5].

Although insulin resistance and T2D have been much studied in the SA population during recent years, few studies have used gold standard methods for the measurement of insulin resistance, and to our knowledge, no previous studies have reported endogenous glucose production (EGP) in SA T2D subjects.

In order to gain insights into possible explanations for the differences in prevalence of T2D between the two populations, we analysed baseline data from a vitamin D intervention trial in subjects with T2D and Nordic (NOR) or SA origin, all living in Oslo, Norway. Insulin sensitivity, glucose and fat oxidation, and insulin secretion was measured. The subjects all had serum levels of 25-hydroxyvitamin D ≤50 nM.

The primary aims of this study were to: 1) Explore possible differences in fasting and clamp hyperinsulinemic glucose metabolism, 2) Explore possible differences in insulin secretion, 3) Explore possible differences in energy expenditure, and 4) Assess possible associations between insulin sensitivity and -secretion, and vitamin D status.

## Methods

### Ethics

All participants gave informed written consent prior to any study related procedure. The study was approved by the South-Eastern Norway Regional Committee for Medical and Health Research Ethics, and conformed to the Declaration of Helsinki.

### Subjects

Sixty-two patients with T2D and 25-hydroxyvitamin D ≤50 nM, were recruited from our out-patient clinic, from general practice, from posters in the hospital lobby and in pharmacies, and from advertisements in local newspapers. Men and women with T2D, from the Oslo area in Norway, above 18 years of age, of Nordic or South Asian origin (born in a South Asian country and/or with both parents of South Asian origin) were eligible, regardless of type of anti-diabetic treatment. Full inclusion and exclusion criteria are displayed in Table 1. In total, 190 patients were screened. Sixty-two patients met with the inclusion criteria and were recruited, and 61 patients underwent initial intra-venous glucose tolerance tests (IVGTT) and clamp procedures. The Nordic group consisted of 40 Norwegians, 2 Danes and 1 Swede. The South Asian group consisted of 11 Pakistanis, 7 Sri Lankans (all Tamil) and 1 Indian. All but one of the South Asian participants were first generation immigrants. One Norwegian patient was excluded due to severe difficulties in getting intravenous access. Table 2 shows important characteristics of the two ethnic groups.

**Table 1:** Inclusion and exclusion criteria.

Inclusion criteria:
**V**itamin D deficiency defined as 25-hydroxyvitamin D <50 nM
**P**atients with type 2 diabetes (negative anti-GAD and anti-IA2), including drug naïve subjects, subjects using oral anti-diabetic medication and subjects on insulin treatment
**H**bA_1c_ <11% (97 mmol/mol) at inclusion
**M**en and women ≥18 years
**N**ordic or South Asian ethnicity (from Pakistan, India, Bangladesh or Sri Lanka)
**A**ntihypertensive medication, lipid lowering drugs, oral contraceptives, hormone replacement therapy, multivitamin supplements and nutritional supplements are allowed
**Exclusion criteria:**
**S**ystolic Blood Pressure ≥160 mmHg or Diastolic Blood Pressure ≥90 mmHg at inclusion
**S**ignificant renal disease or chronic renal impairment, GFR<30 mL/min
**S**ignificant liver disease or ASAT or ALAT >3× upper limit of normal
**M**alignancy during the last five years
**H**ypercalcemia at inclusion or a history of kidney stone disease
**P**regnant or breastfeeding women
**C**hronic inflammatory disease in active phase or long term (>2 weeks) use of systemic corticosteroids last 3 months
**C**ardiovascular disease, defined as myocardial infarction, unstable angina pectoris or stroke, during the last 6 months prior to inclusion
**A**nemia defined as hemoglobin below current reference limits
**B**MI >45 kg/m^2^ or bariatric surgery performed during the last five years
**D**rug or alcohol abuse
**M**ental condition (psychiatric or organic cerebral disease) rendering the subject unable to understand the nature, scope and possible consequences of the study
**A**ny medical condition that in the judgment of the investigator would jeopardize the subject’s safety

Main inclusion and exclusion criteria. GAD: glutamic acid decarboxylase, IA2: protein tyrosine phosphatase, GFR: glomerular filtration rate, ASAT: aspartate amino transferase, ALAT: alanine amino transferase, BMI: body mass index.

**Table 2:** Description of patients.

	NOR	SA	
	n = 43	n = 19	*p*
Sex, males n (%)	28 (65.1%)	9 (47.4%)	*0.263^ b^*
Age, years	58.3±8.3	49.7±9.4	***0.001^a^***
Age at diabetes debut,years	48.7±9.1	40.4±10.4	***0.002^a^***
Diabetes duration, years	9.6±7.0	9.3±5.4	*0.864^a^*
Diabetes medication, n (%):			*0.564^b^*
Lifestyle ± OAD or GLP-1	26 (60.5%)	10 (52.6%)	
Insulin ± OAD	17 (39.5%)	9 (47.4%)	
Diabetes complications,n (%):	20 (46.5%)	8 (42.1%)	*0.788^b^*
Cardiovascular disease	3 (7.0%)	2 (10.5%)	
Nephropathy/microalb.	8 (18.6%)	2 (10.5%)	
Other	17 (39.5%)	7 (36.8%)	

Data are presented as number (percentage) or as mean ± standard deviation. *p*-values from *^a^*Student's t-test or *^b^*Chi square test. OAD: oral antidiabetic agent, GLP-1: Glucagon-like peptid 1 analogue. Microalb.: microalbuminuria. Other complications include ophthalmopathy, neuropathy, diabetic foot, sexual dysfunction and periodontal disease. NOR = Nordic, SA = South Asians.

### Anthropometrics

Height to the nearest 0.1 cm and weight to the nearest 0.1 kg were measured with participants wearing light clothing and no shoes. Waist circumference was assessed with a flexible tape measure with spring scale to ensure equal traction at every measurement, measuring at mid-point between the lowest rib margin and the iliac crest. The body surface area was calculated using Mostellers equation [6]. Fat mass (FM) in kg, percentage total body fat (%TBF), percentage truncal fat (% truncal fat) and fat free body mass (FFM) in kg were measured by dual x-ray absorptiometry (DXA) on a Lunar Prodigy from GE Healthcare.

### IVGTT and Euglycemic Clamp

To enhance comparability of examinations, all patients were asked to stop oral antidiabetic drugs for two days, and insulin for at least 12 hours prior to examination. Patients were also asked to refrain from strenuous physical exercise and alcohol intake during these two days, and to arrive fasting for at least 10 hours, from the night before the examination.

A teflon catheter was placed in a vein at each elbow. All infusions were given in one vein, and all blood samples were drawn from the other vein, which was kept open by a slow infusion of NaCl 0.9%. The arm where blood samples were taken was kept at 37°C by a heating sleeve connected to a thermal control unit (Swetron AB, Veddestad, Sweden), to arterialize blood samples.

We performed an IVGTT followed by a euglycemic, hyperinsulinemic clamp, with estimation of endogenous glucose production (EGP) using the stable isotope dilution method. A primed (170 mg) continuous (1.7 mg/min) infusion of [6,6-^2^H_2_] glucose (Cambridge Isotope Laboratories, Inc., Andover, MA) was maintained throughout the experiment. After a 2-h tracer equilibration period, the IVGTT was performed, with a <1-minute intravenous bolus injection of glucose 500 mg/mL, 0.3 g/kg body weight. Blood samples were drawn for plasma glucose concentration as well as serum insulin and C-peptide determination at −2, 0, 2, 4, 6, 8, 10, 15 and 30 minutes after glucose bolus injection. Immediately following the IVGTT, a euglycemic, hyperinsulinemic clamp was performed using a modification of the method originally described by De Fronzo et al [7]. Human insulin (Actrapid®, Novo Nordisk, Bagsvaerd, Denmark) was diluted in 500 mL NaCl 0.9%, to 300 mU/mL, after having first added 2 mL of the patients own blood, to avoid insulin sticking to the walls of the bag, and 10 mmol KCl. Insulin was infused at a rate of 80 mU/m^2^⋅min, after an initial bolus and 10 minute priming infusion, determined by the patients pre-clamp plasma glucose. The infusion was maintained for 2 ½ hours or more, until at least 30 minutes of stable euglycemia was obtained. When plasma glucose reached euglycemia, a variable infusion of glucose 200 mg/mL enriched with 8 mg/g glucose of [6,6-^2^H_2_]-glucose was continually adjusted to maintain euglycemia.

Plasma glucose was regularly measured on a Presicion Xceed glucometer (Abbott Laboratories. Abbott Park, IL), with five-minute intervals when the patient approached euglycemia. Control measurements at least every 30 minutes were performed on a Y.S.I 2300 STAT analyzer (Yellow Springs Instruments Inc, Yellow Springs, OH). At the end of the clamp, three measurements of serum insulin and fluoride/oxalate-plasma for analysis of [6,6-^2^H_2_]-glucose were taken at ten-minute intervals. The glucose infusion rate (GIR) in µmol/kg FFM⋅min was established.

### IVGTT Calculations of Insulin Secretion

The Acute Insulin Response to glucose (AIRg) was calculated as the incremental area under the curve (AUC) for insulin from time 0–8 minutes and 0–30 minutes.

### Endogenous Glucose Production Calculations

Calculations of endogenous glucose production (EGP) at the end of the basal equilibration period and during clamp euglycemia were performed. Both were steady state for plasma glucose, with only relatively small changes in glucose concentration and tracer enrichment over time. Thus, steady state equations, where rate of appearance equals rate of disappearance, have been applied for the calculation of both EGP and total glucose disposal (TGD) [8], [9]. The EGP in the basal state was calculated as follows: EGP_basal_ = I((E_i_/E_p(basal)_) –1), where I is the rate of [6,6-^2^H_2_]-glucose infusion (µmol/m^2^⋅min), E_i_ is the enrichment of the tracer infusion in moles percent excess (mpe) and E_p(basal)_ is the mean [6,6-^2^H_2_]-glucose enrichment in plasma (mpe) at the end of the basal stabilisation period.

At the end of the euglycemic clamp, TGD was calculated as follows: TGD = ((I ⋅ E_i_+GIR ⋅ E_m_)/E_p(clamp)_) – I, where GIR is the exogenous glucose infusion rate (µmol/m^2^⋅min), E_m_ is the [6,6-^2^H_2_]-glucose enrichment (mpe) in the infused glucose, and E_p(clamp)_ is the mean [6,6-^2^H_2_]-glucose enrichment (mpe) in the plasma samples taken during the last 30 minutes of the clamp euglycemia. The EGP during clamp euglycemia, EGP_clamp_ = TGD – GIR. Between subject and within subject coefficients of variation for plasma glucose levels in clamp steady state were 10.0% and 4.6% respectively.

### Indirect Calorimetry

Indirect calorimetry was performed in 38 of the 43 NOR and 14 of the 19 SA patients, using a Jaeger Oxycon Pro (Erich Jaeger, Viasys Healthcare, Germany) computerized flow-through canopy gas analyzer system. After a 10-minute adaptation period, expired and inspired air was continuously sampled and analyzed for O_2_ and CO_2_ content during a 30 minute steady state period at the end of the basal tracer equilibration period and at the end of the euglycemic clamp. Whole body substrate oxidation was estimated from the mean values of VO_2_ and VCO_2_ measured, and from measurement of urinary nitrogen (urea). Average basal and insulin stimulated glucose and lipid oxidation rates were calculated using Frayn’s equations [10]. Non-oxidative glucose metabolism was calculated as the difference between total body glucose disposal (as determined by the euglycemic clamp with tracer dilution method) and the rate of glucose oxidation (as determined by indirect calorimetry).

### Blood Samples

Full blood glucose was measured by glucose oxidase method (YSI 2300, Yellow Springs, OH), and plasma glucose was calculated (full blood glucose×1.119). HbA_1c_ was measured by HPLC on a Tosoh G7 analyser (Tosoh Corp., Tokyo, Japan), serum insulin and C-peptide were measured using an immunofluorometric assay (DELFIA) from Perkin Elmer Life Sciences (Wallac Oy, Turku, Finland), 25-hydroxyvitamin D was measured on a radioimmunoassay (RIA) kit from DiaSorin (Stillwater, MN). [6,6-^2^H_2_]-glucose was measured by LC-MS/MS, via turbulent flow chromatography (Cohesive technologies RXT1, Franklin, MA) combined with tandem mass spectrometry (Sciex API3000, Applied Biosystems, Foster City, CA) as previously described [11], at the Clinical Metabolomics Core Facility, (Rigshospitalet, Copenhagen, Denmark). Urinary urea was measured by enzymatic-kinetic UV assay on a Roche Modular P analyser.

### Statistical Analysis

Data are presented as mean ± standard deviation or median [inter-quartile range] unless otherwise specified. We analyzed non-normally distributed data log-transformed, or using non-parametric methods, as appropriate. Student’s *t* tests or Mann Whitney U tests were used for comparison of continuous variables between groups, and paired samples *t*-tests were used for within groups analysis of change. For comparison of categorical data between patient groups, the Chi square test for independence was used. Spearman’s correlation coefficients (r_s_) were used. One–way between-groups ANCOVA was performed, with preliminary checks to ensure no violation of the assumptions of normality, linearity, homogeneity of variances and homogeneity of regression slopes. Multiple linear regression analyses were performed, with log-transformation of parameters when needed, to ensure no violation of the assumptions of normality, linearity and homoscedasticity. In regression analyses NOR = 1 and SA = 2. A two-sided p-value <0.05 was deemed significant, and uncorrected p-values are presented. Bonferroni-Holm corrections were performed, showing that p-values <0.01 remained <0.05 after correction. Statistical analyses were performed with SPSS 19.0 for windows (SPSS Inc., Chicago, IL).

## Results

### General Description

Anthropometric and biochemical characteristics by ethnic group are presented in Table 3. The SA subjects were significantly shorter and leaner than the NOR subjects, but had a higher median HbA_1c_, whereas median fasting C-peptide was significantly higher in the NOR group. Despite a higher waist circumference and waist-to-height ratio in the NOR subjects, the SA still had similar percentage total and truncal fat to the NOR group. Adjusting for sex and/or age did not change these results (data not shown).

**Table 3:** Anthropometrical and biochemical characteristics.

	NOR	SA	
	n = 43	n = 19	*p*
Height, cm	173.6±8.8	163.4±8.4	***<0.001***
Weight, kg	100.4 [15.0]	79.1 [15.0]	***<0.001*** a
BMI, kg/m^2^	33.2±4.7	28.7±4.0	***<0.001***
Waist circumference, cm	115.5 [18.0]	100 [10.6]	***<0.001*** a
Waist/Height ratio	65.5±7.2	61.2±6.8	***0.033***
TBF, %	35.7±7.2	34.5±7.6	*0.56*
Truncal fat, %	40.5±5.7	40.1±6.6	*0.82*
FFM, kg	63.8±9.7	50.1±7.7	***<0.001***
FM, kg	33.1 [17.1]	25.0 [10.8]	***<0.001*** a
Fasting plasma glucose, mM	9.1 [4.5]	10.7 [6.4]	*0.08* a
HbA_1c_, %	7.3 [1.6]	8.5 [1.6]	***0.024*** a
Fasting insulin, pM	85.5 [99.0]	68.0 [138.0]	*0.67* a
Fasting C-peptide, pM	1137 [785]	1012 [431]	***0.049*** a
25(OH)vitamin D, nM	40.1±10.3	31.5±14.0	***0.009***

Data are presented as mean ± standard deviation or median [inter-quartile range]. NOR: Nordic, SA: South Asians, BMI: body mass index, TBF: total body fat, FFM: fat free mass, FM: fat mass, 25(OH)vitamin D: 25-hydroxyvitamin D. *p-*values are from Student's *t*-test.

^a^ = Student's *t-*test after Log-transformation.

### Endogenous Glucose Production

**Figure 1:**
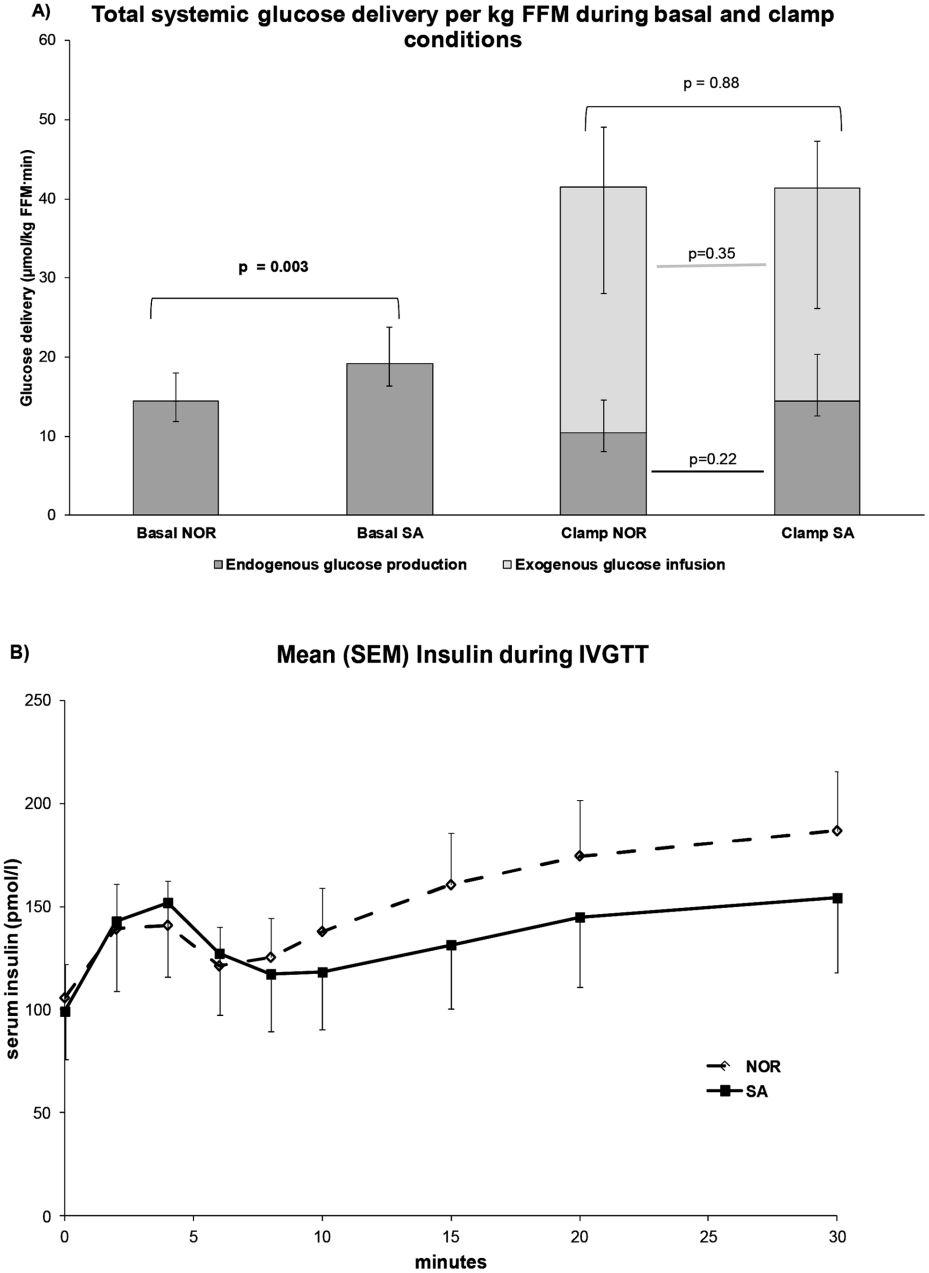
Glucose delivery and insulin secretion during basal and clamp conditions. A) Median [inter-quartile range] values of basal and clamp glucose delivery per kg fat free mass (FFM), both from endogenous glucose production and exogenous glucose infusion. *p*-values from Mann-Whitney U tests. B) Mean (standard error of mean) serum insulin levels during the 30-minute intra-venous glucose tolerance test. NOR = Nordic, SA = South Asians.

EGP_basal_ was significantly higher in the SA than the NOR group, as shown in Table 4 and Figure 1A. This difference remained significant after adjustment for possible confounders, including sex, age, height, weight, BMI, %TBF, FFM, HbA_1c_, fasting C-peptide, or fasting plasma glucose (data not shown). During clamp hyperinsulinemia the EGP was reduced, the ethnic difference in endogenous glucose production (EGP_clamp_) was attenuated, and no longer significant.

**Table 4:** Endogenous glucose production, insulin sensitivity and insulin secretion.

		NORn = 41	SAn = 18	*p*
EGP_basal_	µmol/kg FFM⋅min	14.4 [6.8]	19.1 [9.1]	***0.003***
EGP_clamp_	µmol/kg FFM⋅min	8.9 [6.7]	10.8 [10.4]	*0.216*
EGP_clamp_ %	% of TGD	24.9 [24.3]	38.7 [27.7]	*0.107*
GIR	µmol/kg FFM⋅min	28.9±15.8	24.7±14.6	*0.343*
TGD	µmol/kg FFM⋅min	39.2±17.6	39.1±20.4	*0.990*
Se-insulin_end clamp_	pM	1290 [425]	1270 [1087]	*0.889*
AIRg _0–8 min_	AUC_ 0–8 min_	124 [275]	220 [302]	*0.352*
AIRg _0–30 min_	AUC _0–30 min_	1003 [1505]	852 [1452]	*0.383*
LogAIRg _0–8 min_	LogAUC _0–8 min_	2.15±0.52	2.34±0.44	*0.201*
LogAIRg _0–30 min_	LogAUC _0–30 min_	3.06±0.36	2.98±0.32	*0.425*

Data are presented as mean ± standard deviation or median [inter-quartile range]. *p*-values from Student’s *t*-tests or Mann-Whitney U tests as appropriate. NOR: Nordic, SA: South Asians, EGP: endogenous glucose production, FFM: fat free mass, TGD: total glucose disposal, GIR: glucose infusion rate, AIRg: acute insulin response to glucose, AUC: area under the curve (from 0–8 minutes and 0–30 minutes of the intra-venous glucose tolerance test). For LogAIRg _0–8 min_ n = 36 NOR and 16 SA, and for LogAIRg_0–30 min_ n = 40 NOR and 17 SA.

EGP_clamp_ was detectable in all patients, ranging from 3.4% to 90.6% of the total glucose disposal rate (TGD), with a median of 25.8%. In an effort to find predictors of EGP_clamp_ variation, we performed simple correlations between EGP_clamp_ and parameters which could influence EGP_clamp._ The following parameters correlated to EGP_clamp_ with a *p-value <0.1*: diabetes duration, fasting plasma glucose (FPG), se-insulin at end of clamp, fasting se-C-peptide and HbA_1c_ (Table 5). We then performed an all subsets multiple regression analysis, using logEGP_clamp_ as dependent variable. Log FPG was the only significant parameter to remain, with an unstandardized beta = 0.78, *p = 0.003* and an R^2^ of only 0.15. The separate correlation coefficients in the ethnic subgroups showed differences: FPG and most of the other parameters correlated significantly to EGP_clamp_ only in the NOR group. The correlation between GIR and EGP_clamp_ was neither significant in the total patient group nor in the two separate ethnic subgroups. The correlation between TGD and EGP_clamp_ was significant in the SA subgroup but not the NOR subgroup.

**Table 5:** Correlations to endogenous glucose production during clamp.

		Total patients	NOR	SA
		n = 57	n = 39	n = 18
Diabetes duration	r_s_	0.251	**0.343**	−0.058
	*p*	*0.059*	***0.033***	*0.819*
Fasting plasma glucose	r_s_	**0.420**	**0.524**	0.057
	*p*	***0.001***	***0.001***	*0.823*
Serum insulin at end of clamp	r_s_	**−0.295**	−0.187	−0.387
	*p*	***0.026***	*0.254*	*0.113*
Fasting serum C-peptide	r_s_	**−0.272**	**−0.320**	−0.034
	*p*	***0.040***	***0.047***	*0.926*
HbA_1c_	r_s_	**0.335**	**0.338**	0.101
	*p*	***0.011***	***0.035***	*0.689*
Exogenous glucose infusion rate	r_s_	−0.035	−0.116	0.228
	*p*	*0.798*	*0.480*	*0.363*
Total glucose disposal	r_s_	**0.318**	0.156	**0.591**
	*p*	***0.016***	*0.342*	***0.010***

Data are presented as Spearman’s correlation coefficients (r_s_) with corresponding *p*-values. Significant correlations in bold. NOR: Nordic, SA: South Asians.

### Insulin Sensitivity

There was no significant ethnic difference in insulin sensitivity expressed as the TGD in µmol/kgFFM⋅min (Table 4, Figure 1A). After adjusting TGD for log EGP_clamp_ (beta = 28.4, *p = 0.001*) and log waist circumference (beta = −114.7, *p = 0.028),* in a multiple regression analysis, ethnicity came closer to significance (beta = −9.1, *p = 0.111)*. Further adjusting for age *(p = 0.84)* or sex *(p = 0.97)* was not significant.

### Insulin Secretion

All but seven of the 60 subjects where an IVGTT was performed had some preserved first phase insulin secretion, (increased incremental AUC_0–8_), and two thirds of the patients displayed an AUC_0–8_>100 pM. Insulin secretion (AIRg) did not differ significantly between the two ethnicities (Table 4, Figure 1B). After adjusting for HbA_1c_ in a multiple regression analysis to account for possible glucose toxicity, there was a non-significant trend towards higher insulin secretion in the SA group (beta = 0.30, *p = 0.052*, model significance: *p = 0.030)*. LogAUC_0–8_ was the dependent variable and ethnicity and log HbA_1c_ (beta = −2.27, *p = 0.02*) were independent variables. Further adjusting for age *(p = 0.39)* and sex *(p = 0.51)* was not statistically significant. When measured as the AUC_0–30_, insulin secretion did not differ between the two ethnic groups, neither before nor after adjustment for HbA_1c_, age and/or sex. A longitudinal analysis of AUC for insulin during the total 30 minutes of IVGTT did not show any significant ethnic difference either (Figure 1B).

### Glucose and Fat Oxidation and Non-oxidative Glucose Metabolism

**Figure 2:**
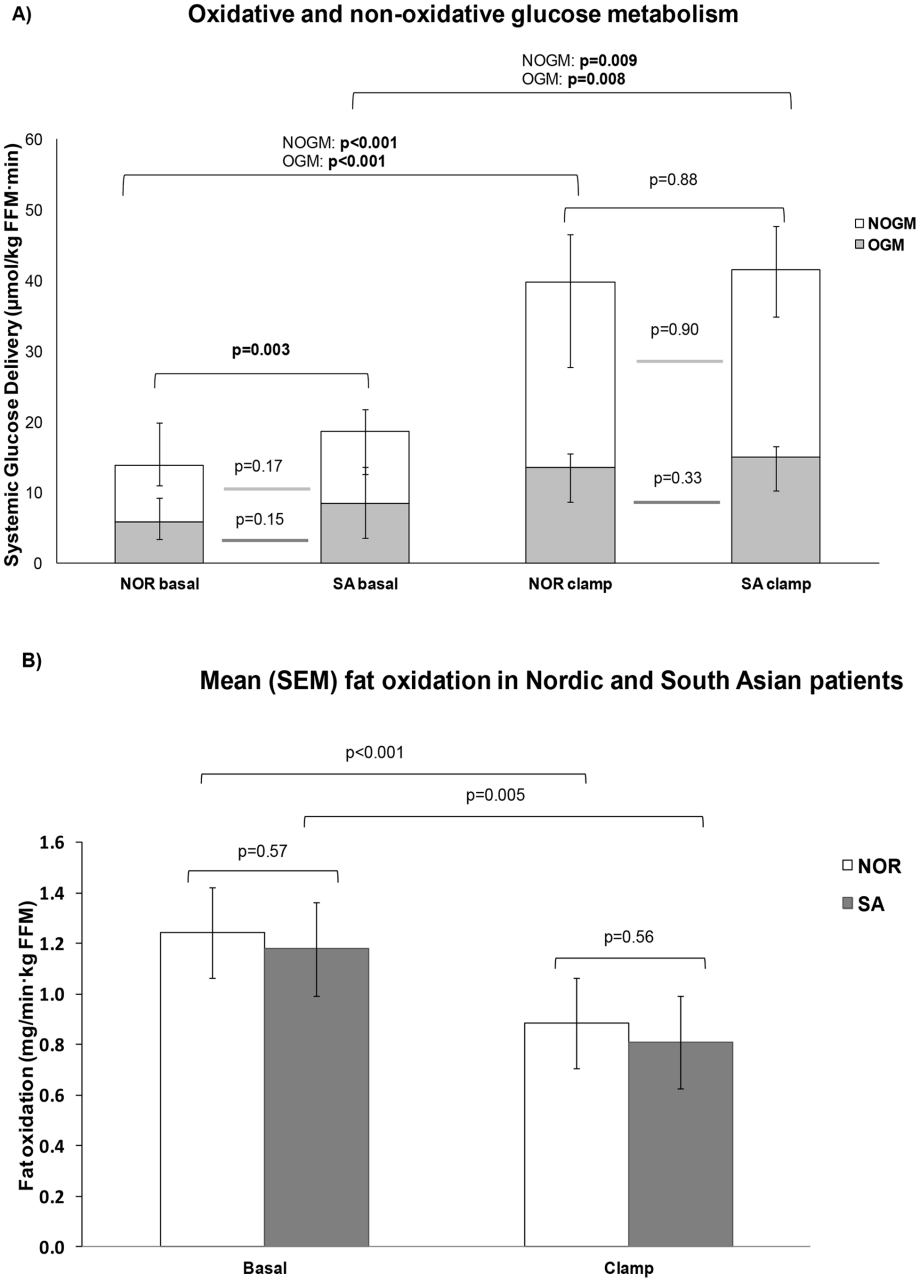
Glucose and fat metabolism in Nordic and South Asian subjects with type 2 diabetes. A) Glucose metabolism per kg fat free mass (FFM). Median [inter-quartile range] values of basal and clamp glucose delivery, both from non-oxidative glucose metabolism (NOGM) and oxidative glucose metabolism (OGM) B) Fat oxidation per kg fat free mass (FFM). Mean (standard error of mean) values in basal and clamp conditions. NOR = Nordic, SA = South Asians. Comparisons beween ethnic groups are Student’s *t*-tests or Mann-Whitney U tests as appropriate. Comparisons between basal and clamp values are paired samples *t*-tests, after log-transformation where appropriate.

Figure 2 displays glucose and fat metabolism in peripheral tissues in the basal fasting and the hyperinsulinemic clamp state, measured by indirect calorimetry. Figure 2A demonstrates that higher endogenous glucose production in SA leads to increases in both oxidative and non-oxidative metabolism in peripheral tissues. This figure also demonstrates the higher non-oxidative than oxidative metabolism in the basal state in both ethnic groups, and that non-oxidative glucose metabolism increases more than oxidative in the clamp hyperinsulinemic state in both ethnicities.

Basal fat oxidation measured per kg fat free body mass was similar in the two ethnic groups (Table 6, Figure 2B). Fat oxidation decreased during clamp hyperinsulinemia, as glucose metabolism increased. These changes were similar in the two groups.

**Table 6:** Basal and Clamp Indirect Calorimetry.

		NORn = 38	SAn = 14	*p*
Basal glucose oxidation	µmol/kg FFM⋅min	6.5±4.5	8.5±4.0	*0.151*
Basal non-oxidative glucose consumption	µmol/kg FFM⋅min	9.0±5.9	12.0±9.1	*0.167*
Basal fat oxidation	mg/kgFFM⋅min	1.24±0.37	1.18±0.36	*0.572*
Clamp glucose oxidation	µmol/kg FFM⋅min	12.7±4.6	14.2±6.7	*0.331*
Clamp non-oxidative glucose consumption	µmol/kg FFM⋅min	26.3 [17.6]	26.6 [29.5]	*0.897*
Clamp fat oxidation	mg/kgFFM⋅min	0.89±0.38	0.81±0.50	*0.557*
REE	kJ/day	7465±1202	6104±1214	***0.001***
EE_clamp_	kJ/day	7750±1315	6263±1139	***<0.001***
RQ_basal_		0.79±0.05	0.80±0.04	*0.201*
RQ_clamp_		0.84±0.05	0.86±0.06	*0.440*
ΔRQ		0.052±0.035	0.053±0.062	*0.994*

Data are presented as mean ± standard deviation or median [inter-quartile range]. *p*-values from Student’s *t*-tests or Mann-Whitney U tests as appropriate. NOR: Nordic, SA: South Asians, FFM: fat free mass, REE: resting energy expenditure, EE_clamp_: energy expenditure during clamp, RQ: respiratory quotient, ΔRQ: change in respiratory quotient from basal to clamp conditions.

### Basal and Clamp Energy Expenditure

The mean, unadjusted resting energy expenditure (REE) in kJ/day, estimated by indirect calorimetry, was higher in the NOR than in the SA patients (Table 6). However, after adjustment for FFM, FM, age and sex in a one-way ANCOVA analysis, the ethnic difference was attenuated, and no longer significant (*p = 0.51*), with adjusted mean (SEM) values of 7155 (121) kJ/day in NOR and 6954 (239) kJ/day in SA patients.

REE correlated highly with basal fat oxidation (r_s_ = 0.48, *p = 0.002* in NOR and 0.64, *p = 0.014* in SA patients), but not with basal glucose oxidation (r_s_ = −0.06, *p = 0.73*, and −0.10, *p = 0.75*, respectively), or non-oxidative glucose metabolism (r_s_ = −0.16, *p = 0.36*, and r_s_ = 0.40, *p = 0.16* respectively), although SAs showed a stronger correlation between REE and non-oxidative glucose metabolism than the NOR group.

The positive correlation between REE and EGP_basal_ tended to be stronger in SA (r_s_ = 0.53, *p = 0.051*), compared to the NOR subjects (r_s_ = −0.18, *p = 0.28*). The correlation between EE_clamp_ and EGP_clamp_ was also stronger in SA (r_s = _0.50, *p = 0.082*), than in NOR subjects (r_s_ = −0.06, *p = 0.74*). Energy expenditure increased significantly during clamp (EE_clamp)_ in the NOR patients (*p = 0.003*), but not in the SA patients (*p = 0.28*). The respiratory quotient (RQ) increased significantly from basal to clamp value in both NOR (*p<0.001*) and SA subjects (*p = 0.008*) (Table 6). This change (ΔRQ) was similar in the two groups.

### Relation between Insulin Sensitivity, Insulin Secretion and Vitamin D

Median serum 25-hydroxyvitamin D in the SA group was significantly lower than in the NOR group (table 3). We found no significant correlations between 25-hydroxyvitamin D levels and insulin sensitivity or insulin secretion, neither in the two ethnic groups examined separately, nor in the total cohort.

## Discussion

In this study we examined ethnic differences in glucose and fat metabolism and energy expenditure in basal and clamp hyperinsulinemic conditions in subjects with T2D, of SA or NOR ethnicity, living in Oslo, Norway. We found evidence of ethnic differences in fasting endogenous glucose production, and indications of possible differences in the choice of substrates for energy expenditure both in basal and clamp conditions.

The concept of ethnicity and ethnic groups is complex, consisting of both socio-cultural and biological components that are not clearly defined [12]. The term South Asian ethnicity is often used, although the South Asian region is diverse, with several countries (Pakistan, India, Bangladesh and Sri Lanka), and differences in culture, religion and diet. In diabetes research, using the term South Asian can nonetheless be justified, in view of the fact that the high prevalence of diabetes and increased insulin resistance is present in the whole region [13], particularly in urban areas, and also after migration to Western countries [1].

This study shows a significantly higher fasting EGP in SA compared to NOR patients, which was not explained by any of the examined possible confounding factors. During clamp hyperinsulinemia the EGP was lowered, and the ethnic difference was attenuated. Even so, EGP_clamp_ was still not negligible, and constituted almost 40% of TGD in SA and 25% in NOR patients. Hyperinsulinemia during clamp is often said to suppress EGP almost entirely [14], [15], and euglycemic clamp studies are still frequently performed without the measurement of endogenous glucose production [16], [17]. However, several authors have demonstrated that EGP_clamp_ persists [18]–[20]. We here present further evidence that EGP_clamp_ can be substantial in type 2 diabetic patients, even with serum insulin concentrations during clamp as high as 1000–1500 pM. This finding underscores the importance of controlling for hepatic glucose production during clamp studies.

Measuring EGP via the isotope tracer dilution method is both time consuming and costly. In an attempt to find predictors for the estimation of EGP_clamp_ from variables that are easier to measure, we looked at a group of variables which correlated with EGP_clamp_. Only FPG remained significantly related to EGP_clamp_ in regression analyses, and it explained only 15% of EGP_clamp_ variation in the whole patient group. When looking at the correlations in the separate ethnic subgroups, most variables only correlated significantly in the NOR group. The exogenous glucose infusion rate did not correlate to EGP_clamp_ at all. In the SA group, the only significant correlation was between EGP_clamp_ and TGD, merely reflecting the high percentage of EGP_clamp_ in TGD in this group. We therefore suggest that measuring EGP_clamp_, in addition to the exogenous glucose infusion rate, is essential for correct estimation of total glucose disposal rate.

Some ethnic groups residing in tropical climates, including SA, have previously been shown to have lower REE than Westerners [21], however, several authors have advocated the need for adjusting REE for fat free mass (FFM) and fat mass (FM), as well as age and sex [4], [22]. The lower REE in SA is in this way shown to be due to differences in body composition and not due to ethnicity per se. We find it to be the case also in our study. Our two ethnic groups display clear differences in body composition, and adjusting for these differences, mainly FFM, attenuates the ethnic difference seen in REE. This, however brings us back to the complex concept and definition of ethnicity, whence it can also be argued that lower FFM is an ethnic characteristic of South Asians. This has been described in other studies [23], [24].

In basal, resting conditions, energy production is for the most part derived from lipids, and less from carbohydrates [25]. This is also reflected in our study, by the highly significant correlation between REE and fat oxidation in both ethnicities. In the SA subgroup, however, there is also a near-significant correlation between REE and EGP_basal_. This could signify increased use of carbohydrates as energy substrate in the fasting state in SA, to such an extent that it becomes important for the total REE. However, we did not find any ethnic differences in basal RQ or in ΔRQ from fasting to clamp hyperinsulinemic conditions, that would have clearly indicated an ethnic difference in metabolic flexibility. The values of basal RQ and ΔRQ in our subjects were comparable to the group with diabetes in the recently published study by van de Weijer [26]. In that study, they also demonstrated that insulin stimulated RQ is mainly dependent on glucose disposal rates, which in our study are similar in the two groups.

Our NOR participants are significantly more obese than the SA, which could in part explain why there is no obvious ethnic difference in TGD. After adjustment for difference in waist circumference, as well as the endogenous glucose production, the ethnic difference in TGD came closer to significance.

In our study we found a non-significant trend towards higher both oxidative and non-oxidative glucose metabolism in SA compared to NOR subjects in the basal, post-absorptive state. The total EGP_basal_ was significantly higher. This points towards a higher degree of hepatic insulin resistance in SA. One could speculate that a possible higher basal oxidative glucose metabolism in muscle, triggered by increased substrate availability from fasting hyperglycemia, leads to less use of lipids as energy substrate, again leading to lipid accumulation and further aggravation of the hepatic insulin resistance, as described in the metabolic inflexibility hypothesis [3]. In post-absorptive conditions skeletal muscle contribution to total metabolism is modest [3]. A possible ethnic difference in muscle metabolism could thus have been masked.

The hepatic insulin resistance could also initially have been caused by increased lipid storage in the liver due to adipose tissue overflow [2]. Percentage truncal fat was similar in our two ethnic groups, although the NOR group was significantly more obese. In a previous study we found that even though a group of Pakistani and Norwegian subjects with T2D had similar abdominal adipose tissue distribution, the visceral adipose tissue was more metabolically active in the Pakistani subjects (Wium C, Eggesbo HB, Ueland T et al, 2013, unpublished data). An increased supply of NEFA from visceral adipose tissue to the liver would, in addition to the effect of increasing the insulin resistance, constitute a source of substrate for gluconeogenesis.

Gluconeogenesis is known to be increased in subjects with T2D, being in large part responsible for the increased post-absorptive EGP. When using [6,6-^2^H_2_] glucose as tracer, Cori cycling is included in the estimation of the total glucose disposal, and has been shown to be increased 25% in T2D in general [27]. It is possible that the increase in non-oxidative glucose metabolism in SA in large part corresponds to increased Cori cycling, due to substrate availability through hyperglycemia in tissues, with lactic acid production in muscle or other tissues by anaerobic glycolysis, then transport back to the liver and re-use as substrate in gluconeogenesis, creating a vicious circle.

The data in this study are baseline results from a vitamin D intervention trial. It was therefore of interest to look for associations between baseline 25-hydroxyvitamin D levels and measures of insulin sensitivity and insulin secretion. We found significantly lower median 25-hydroxyvitamin D levels in the SA group. Could differences in vitamin D status explain some of the ethnic differences in glucose metabolism? Several epidemiological studies have in recent years shown a relationship between vitamin D and diabetes [28], [29], the metabolic syndrome [28], insulin resistance [30], [31], and some studies also with insulin secretion [32]. However, in most of the published studies that report significant associations, or an effect of vitamin D intervention, the primary end points have been surrogate markers based on fasting blood values, like the HOMA indices [28], [33], [34]. The few studies using more sophisticated methods, like OGTT, IVGTT or clamps have usually not been able to show similar significant relationships [35]–[39]. We did not find any association between levels of 25-hydroxyvitamin D and TGD or AIRg. The question therefore still remains whether there is a genuine and causal relationship between vitamin D and diabetes, and we have to await results from randomised, controlled trials.

A strength of this study was the use of gold standard methods such as the euglycemic clamp with tracer dilution, coupled with indirect calorimetry. This enabled us to measure both fasting endogenous glucose production and insulin sensitivity, as well as carbohydrate and fat metabolism, both in the basal and hyperinsulinemic state. To our knowledge, this has not been reported in SA subjects with T2D previously. The patients were included by a large variety of approaches, with wide inclusion criteria, the main restriction being the 25-hydroxyvitamin D levels ≤50 nM, with the aim of securing broad representativeness. The following limitations must be noted: The data presented here are cross-sectional. No efforts towards matching of the two ethnic groups at baseline were made. The inclusion of SA patients in the study proved challenging, hence the SA group was limited in size, increasing the risk of Type II statistical errors. Further studies in a larger group of patients, will therefore be necessary to confirm some of the findings that are still uncertain in our study. We selected only subjects with low levels of vitamin D, and our results therefore cannot be generalized to the whole population of subjects with T2D, although low vitamin D levels are common i T2D. Nevertheless, we did not find any correlation between 25-hydroxyvitamin D levels and insulin sensitivity or insulin secretion. This is an exploratory study, and we have judged it appropriate not to show p-values corrected for multiple testing. Bonferroni-Holm corrections were performed, showing that p-values <0.01 remained significant. However, due to the high risk of missing a genuine difference that is clinically significant, we still focus on the non-corrected tests [40]. Hence, there is also a risk of reporting p-values <0.05 by chance.

## Conclusions

We have demonstrated higher basal EGP in SA patients with established T2D. Clamp EGP can be substantial in patients with established T2D, and cannot be estimated from the surrogate markers measured. We found no ethnic difference in insulin sensitivity or in first phase insulin secretion. Findings of near significant correlations between REE, EE_clamp_ and EGP in the SA group only, might indicate increased post-absorptive glucose metabolism in the SA group, at the expense of lipid metabolism, but these results have to be confirmed in larger studies. Finally, we found no indication of any relation between vitamin D and insulin sensitivity and secretion.
